# Genome-Wide Analysis of Simple Sequence Repeats in Cabbage (*Brassica oleracea* L.)

**DOI:** 10.3389/fpls.2021.726084

**Published:** 2021-12-09

**Authors:** Yuanyuan Xu, Miaomiao Xing, Lixiao Song, Jiyong Yan, Wenjiang Lu, Aisong Zeng

**Affiliations:** Jiangsu Key Laboratory for Horticultural Crop Genetic Improvement, Institute of Vegetable Crops, Jiangsu Academy of Agricultural Sciences, Nanjing, China

**Keywords:** cabbage, genome, SSR, molecular makers, genetic diversity, manual cultivar identification diagram

## Abstract

Cabbage (*Brassica oleracea* L. var. *capitata*) accounts for a critical vegetable crop belonging to Brassicaceae family, and it has been extensively planted worldwide. Simple sequence repeats (SSRs), the markers with high polymorphism and co-dominance degrees, offer a crucial genetic research resource. The current work identified totally 64,546 perfect and 93,724 imperfect SSR motifs in the genome of the cabbage ‘TO1000.’ Then, we divided SSRs based on the respective overall length and repeat number into different linkage groups. Later, we characterized cabbage genomes from the perspectives of motif length, motif-type classified and SSR level, and compared them across cruciferous genomes. Furthermore, a large set of 64,546 primer pairs were successfully identified, which generated altogether 1,113 SSR primers, including 916 (82.3%) exhibiting repeated and stable amplification. In addition, there were 32 informative SSR markers screened, which might decide 32 cabbage genotypes for their genetic diversity, with level of polymorphism information of 0.14–0.88. Cultivars were efficiently identified by the new strategy designating manual diagram for identifying cultivars. Lastly, 32 cabbage accessions were clearly separately by five Bol-SSR markers. Besides, we verified whether such SSRs were available and transferable in 10 Brassicaceae relatives. Based on the above findings, those genomic SSR markers identified in the present work may facilitate cabbage research, which lay a certain foundation for further gene tagging and genetic linkage analyses, like marker-assisted selection, genetic mapping, as well as comparative genomic analysis.

## Introduction

Molecular markers used for research change from enzyme-based to DNA-based ones at present; moreover, numerous DNA markers systems are constructed. (Zhang et al., 2016; Ikten et al., 2019). Simple sequence repeats (SSRs), which are also referred to as microsatellites, represent the tandem repeat sequences containing 2–6 nucleotides (nt) short units and are usually seen in eukaryotic and prokaryotic genomes. SSRs are identified to be the preferred option for different research (Wang et al., 2021; Zhang et al., 2021). SSR markers are highly variable, abundant, reproducible, and transferrable, with co-dominant and multi-allelic inheritance; as a result, they are the precious and creditable approaches to carry out gene tapping, genetic mapping, comparative mapping, and genetic diversity analyses on plant species (Li et al., 2012; Silva et al., 2013). Generally, SSR markers are developed dependent on SSR motifs as well as the corresponding flanking sequences, and they may be separated from non-coding nt sequences or conserved coding regions in each higher organism (Sraphet et al., 2011; Xu et al., 2019). Cross-species amplification has been conducted to discover SSR markers that can be used in plant research, and it is related to the selection of genomic libraries or SSR-abundant cDNA and the search of open databases (Yang et al., 2020). At present, the whole-genome sequence for one specific species can be available, which allows to identify and develop SSR markers at genome-wide level (Karci et al., 2020). Notably, the emergence of next-generation sequencing (NGS) technology has reduced the time and cost necessary to carry out whole-genome sequencing (WGS) for plant species (Portis et al., 2018). Genomes make it possible for the development of numerous SSR markers for assessing genetic variations in germplasms and cultivars, identifying quantitative trait loci and genes for the control of traits with economic importance, develop molecular genetics and physical maps and assist in breeding for crop improvement (Cui et al., 2017; Xue et al., 2018). SSR markers have been identified in the whole-genome of diverse living bodies, such as human beings, insects, marine animals, plants with economic value and medicinal fungi (Gil et al., 2017; Liu et al., 2018, 2019). Nevertheless, with the exception of Chinese cabbage, eggplant and cucumber, research on key vegetable species like cabbage in this field is lacking.

Cabbage (*Brassica oleracea* L., 2*n*=18) is one of the most critical cruciferous vegetables that is widely cultivated all over the world. As a kind of vegetable, cabbage has been extensively consumed throughout the world because it contains favorable components for human health (Lv et al., 2014; Cai et al., 2020). Some molecular markers are used within cabbage, like sequence-related amplified polymorphism (SRAP), universal random amplified polymorphic DNA (RAPD), restriction fragment length polymorphism and amplified fragment length polymorphism (AFLP) markers (Ikten et al., 2019). Currently, informative molecular markers have emerged, which offer precious knowledge for genomic and genetic research on cabbage (Gadaleta et al., 2012; Zhong et al., 2017). However, regardless of the development of SSR markers, those developed from cabbage reference genome are lacking compared with those in other crops. There are inadequate SSR markers to construct linkage mapping or association research on cabbage (Lee et al., 2015; Lv et al., 2017). Additionally, prior works that develop SSR markers for cabbage mostly focus on screening SSR markers from public databases or SSR-abundant libraries (Taheri et al., 2018). The plenty of RNA-Seq data have contributed to *in silico* generation, but there is no available comprehensive analysis on SSRs within cabbage genome, even though related information becomes accessible recently. Moreover, SSR loci have been increasingly utilized for the development of molecular markers that can be applied in genetic analysis, like genome assembly, positional cloning, diversity assessment, as well as breeding activities like marker-assisted selection (MAS), but they are not used or detected in further research.

The release of reference genome of cabbage has produced numerous sequences, which are adopted to develop and identify cabbage SSR markers (Cai et al., 2020; Lv et al., 2020). Thus, the present work focused on identifying SSRs at genome-wide level in the cabbage line TO1000, a homozygous doubled haploid, and evaluate them for marker development.

A large set of 64,546 primer pairs was successfully identified in the cabbage genome. Among these, there were altogether 1,113 SSR primers prepared, and a subset of 916 (82.3%) pairs of primers could be stably and repeatably amplified. Moreover, according to the genetic diversity analyses on 32 cabbage genotypes, we identified a set of 32 SSR markers. By adopting the manual cultivar identification diagram (MCID), it was possible to rapidly distinguish cabbage genotypes by combining 5 SSR primers, the novel strategy enabling the practical and referable application of molecular markers and morphological descriptors. Additionally, SSRs detected within the conserved coding regions were highly available and transferable among 10 relevant species of cruciferous crops; as a result, they were conductive to comparative analysis on relatives belonging to Brassicaceae family. Data on SSR markers contribute to the rapid enrichment of functional molecular markers closely associated with expressed regions within cabbage genes, which show high value in the comparative genomic analysis and genetic mapping of cabbage.

## Materials and Methods

### Plant Materials

A total of 32 cabbage genotypes (Supplementary Table S1) that had diverse morphologies and origins were chosen to analyze the genetic diversity and identify the cultivars, besides, 10 relatives were also chosen from Brassicaceae to study the transferability.

### SSR Content of the Cabbage Genome

This study obtained the high-quality cabbage genome within the homozygous doubled haploid “TO1000,” in the format of FASTA (freely accessible at www.ncbi.nlm.nih.gov/genome/10901). Thereafter, we cut 9 pseudomolecules that stood for part of chromosomal sequences in every species, together with those unmapped scaffolds, to small pieces by adopting SciRoKo tool.^1^ Later, SciRoKo SSR-search module was adopted for the *in silico* identification of imperfect, perfect, and compound SSRs. Search queries were specified as at least 4 repetitions and at least 15nt in length. Perfect SSR was defined as a sequence in which one motif was repeated for 4 times (4–6nt motif), 5 times (3nt), 8 times (2nt), and 15 times (1nt), with just one mismatch. As for compound repeats, we set the maximal length of default interruption (spacer) as 100bp. Thereafter, Bedtools was adopted to match those coordinates (start/end positions) for every SSR with gene space, for the sake of intersecting with default parameters under the left outer join option. A repeat was called an SSR when there was one or more than 1nt in the overlap. GO analysis was conducted to define possible gene function carrying one or more SSRs. The enriched GO terms were examined through analyzing SSRs set in genome-wide GO annotation dataset by R ClusterProfiler (v. 3.6.0) package, collected upon the thresholds of false discovery rate<0.01 and values of *p*<e^−5^, and visualized using Cytoscape v. 3.7.1.

### Collection of Genomic Sequences From Different Cruciferous Crops

To compare, we obtained genome sequences for homozygous doubled haploid cabbage “TO1000,” and full-genome sequences for 11 additional plant cultivars in Brassicaceae family, including *Brassica napus*, *Brassica rapa* subsp. *pekinensis*, *Arabidopsis thaliana*, *Brassica cretica, Raphanus sativus*, *Brassica nigra, Brassica juncea*, *Camelina sativa*, *Capsella bursa-pastoris*, *Eutrema yunnanense*, *Barbarea vulgaris*, and *B. oleracea*, based on open database. Then, we performed the above-mentioned process to scan whether perfect SSRs existed. Supplementary Table S2 displays the sources of all the full-genome sequences.

### SSR Identification and Primer Design

SSRs were identified in whole-genome data of cabbage using the MISA package. The SSR motif length was restrained to 1–6bp, which was in consistence with mononucleotides (Mono-), dinucleotides (Di-), trinucleotides (Tri-), tetranucleotides (Tetra-), pentanucleotides (Penta-), and hexanucleotides (Hexa-), separately. Search standards were the same as those in previous study (Cheng et al., 2016). In addition, primers were designed by adopting Perl scripts p3_in.pl./p3_out.pl.^2^ and Primer3 primer modeling software,^3^ and SSR search findings were used to be the input.

Conditions to select primers were shown below, primer size, 18–27bp (optimal, 20bp); melting temperature of primer (*T*m), 57.0–63.0°C (optimal, 60°C), primer GC level, 40–60% (best, 50%) and product size, 100–500bp (optimal, 250bp). Each of the primer pairs designed was later aligned against the ‘TO1000’ cabbage reference genome. We defined unique primer pairs as those whose reverse and forward primers showed unique alignment to reference genome with a 100% match rate.

### DNA Extraction, PCR Amplification and Detection

We chose altogether 48 primer pairs at random for better validating amplification of the particular SSR primer set identified in the present work (Supplementary Table S3). Genomic DNA (gNDA) fragments of ‘QBYS’ and ‘QBJF’ cabbage lines were amplified by using every pair of primers. The CTAB protocol after modification (Liu et al., 2003) was also employed to extract gDNA from the young leaf samples in 32 cabbage accessions of diverse origins and in 10 relevant *Brassica* species.

The 20μl volume was prepared for every PCR procedure, including template DNA (10ng), MgCl_2_ (2.0mm), dNTPs (0.2mm), respective primers (0.1μm) and Taq DNA polymerase (0.5U, TaKaRa Bio Inc., Dalian, China). The reaction procedure was as follows, 3min of initial denaturation under 94°C; 50s under 94°C, 50s under 56°C, as well as 1min under 72°C for 35cycles; final 10min of extension under 72°C. Later, 8.0% PAGE was conducted to separate SSR primers-amplified products for 2–2.5h at 160V, while rapid silver staining (Liu et al., 2008) was performed for visualization.

The AxyPrep DNA gel extraction kit (Axygen Bio Inc., Hangzhou, China) was utilized to recover part of amplified products with desirable size from PAGE gels. Meanwhile, T-A cloning kit (TaKaRa) was adopted for cloning those products extracted, whereas ABI 3730 (Applied Biosystems, United States) was adopted for sequencing the positive clones at Beijing Genomics Institute (BGI Shenzhen, China).

### Survey of Polymorphism and Genetic Diversity Analysis

In order to further estimate the application of these SSR markers and validate the polymorphism of these loci, 32 diverse cabbage cultivars were selected and classified according to the predicted genetic distance (Supplementary Table S1). For SSR markers, we calculated their polymorphic information content (PIC) values by using Power Marker v. 3.0 (Liu and Muse, 2005). Genetic similarity coefficients across the diverse accessions were calculated using NTSYS-pc software SIMQUAL program using the 0–1 data matrix. Moreover, dendrograms were constructed by applying NTSYS-pc software SAHN module *via* the unweighted pair-group method with arithmetic averages (UPGMA; Rohlf, 2000; Kumar et al., 2001).

Furthermore, to facilitate the efficient use of primers and enable them to be easily operated, a strategy designated MCID was adopted, where cultivars were identified by the manual scoring and selection of certain bands (Wang et al., 2011; Korir et al., 2013; Zhai et al., 2014). We distinguished the 32 cabbage genotypes clearly according to other SSR markers used with certain band sizes. In addition, for assessing SSR markers for their amplification efficiency and transferability, we amplified 24 primer pairs of SSRs in 10 relevant crop species belonging to Brassicaceae family by adopting the above-mentioned PCR conditions.

## Results

### The SSR Content of the Cabbage Genome and Cross-Species Comparison

Altogether 64,546 perfect SSR motifs (132.01 SSR/Mb) were identified from the 0.5 Gb in genomic sequence of cabbage, including 3,338 compound SSRs (Table 1). In addition, there were 93,724 imperfect SSR motifs (Table 2). Then, we compared SSRs distribution and level between the ‘TO1000’ cabbage genomic sequence and 11 additional genomes of related plant species to varying levels (the sequence was 5.5 Gb in length, about 0.8 million SSRs). Later, the related information was obtained based on the databases (Supplementary Table S2). The number of perfect SSRs found in the *B. oleracea* genome was similar to those of *B. cretica* (65,262), *E. yunnanense* (53,260), *B. nigra* (52,117), *R. sativus* (49,605), *C. bursa-pastoris* (46,394), and *B. rapa* (42,656). The *B. oleracea* genome was also found to contain almost four times as many perfect microsatellites as that of *A. thaliana* (17,225), and twice the number compared with *B. vulgaris* (34,939). However, it contained only half of those in *C. sativa* (135,740), *B. napus* (123,212), and *B. juncea* (104,035). The cumulative length of the full collection of cabbage SSRs was 1.4 Mbp, which comprises 0.29% of the assembled genome. The same percentage was found in *A. thaliana* (0.29%) and radish (0.29%) but considerably lower than that found in *C. sativa* and *B. vulgaris* (0.48% and 0.40, separately). Compound SSRs represented 5.17% of the cabbage perfect SSRs, which only exceeded those of *A. thaliana, B. cretica* and radish (Table 1).

**Table 1:** A comparative survey of perfect Simple sequence repeats (SSRs) across 12 analyzed genome sequences.

Genome	Analyzed sequences (Mbp)	Perfect SSRs				Compound SSRs	
		Count	Density (SSRs/Mbp)	Cumulative (Mbp)	Cumulative (%)	Count	%
*Brassica napus* L.	976.191	123,212	126.22	2.70	0.28	7,297	5.92
*Brassica rapa* subsp. pekinensis	284.129	42,656	150.13	1.05	0.37	2,223	5.21
*Arabidopsis thaliana* (L.)	119.669	17,225	143.94	0.35	0.29	737	4.28
*Brassica cretica*	412.521	65,262	158.20	1.33	0.32	1972	3.02
*Raphanus sativus* L.	402.328	49,605	123.29	1.15	0.29	2,508	5.06
*Brassica nigra*	402.145	52,117	129.60	1.24	0.31	2,932	5.63
*Brassica juncea* var. tumida	954.861	104,035	108.95	2.22	0.23	9,970	9.58
*Camelina sativa*	641.356	135,740	211.65	3.06	0.48	8,845	6.52
*Capsella bursa-pastoris*	268.431	46,394	172.83	0.93	0.35	2,479	5.34
*Eutrema yunnanense*	415.364	53,260	128.22	1.10	0.26	3,337	6.27
*Barbarea vulgaris*	167.352	34,939	208.78	0.67	0.40	2,764	7.91
*Brassica oleracea*	488.954	64,546	132.01	1.40	0.29	3,338	5.17

**Table 2:** Variation in the repeat length among genomic cabbage perfect and imperfect SSRs.

SSR type	Perfect motif							Imperfect motif		
	Kinds	Count	%	Density (SSRs/Mbp)	Cumulative (Mbp)	Cumulative (%)	Mean repeat Number	Count	%	Density (SSRs/Mbp)
Mono-	2	18,876	29.24	38.60	0.36	25.79	19.1	19,916	21.25	40.73
Di-	4	26,425	40.94	54.04	0.64	45.44	11.8	31,612	33.73	64.65
Tri-	10	12,253	18.98	25.06	0.25	17.70	6.5	13,061	13.94	26.71
Tetra-	31	4,433	6.87	9.07	0.08	6.00	4.5	8,235	8.79	16.84
Penta-	80	1,505	2.33	3.08	0.03	2.48	4.4	13,952	14.89	28.53
Hexa-	204	1,054	1.63	2.16	0.03	2.44	5.1	6,948	7.41	14.21
Total/mean	331	64,546	100.00	132.01	1.40	100.00	12.1	93,724	100.00	191.68

### Characterization of the SSR Motifs by Different Lengths and Repeats

**Figure 1:**
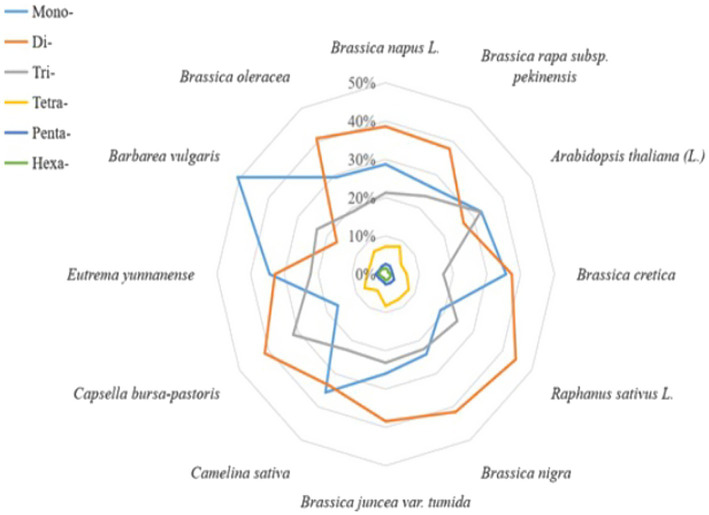
Distribution of SSR repeated motif length across 12 cruciferous genomes.

The cabbage SSR motifs that predominated were the Di- and Mono- (40.9 and 29.2% of all the SSRs, respectively, with densities of 54.04 and 38.60 SSRs/Mbp, respectively), with smaller proportions of trinucleotides (25.06%) and tetranucleotides (6.87%); the penta- and hexanucleotide repeats contributed <5% (Table 2). Dinucleotide sequences played dominant roles, which constituted 0.64 Mbp (45.44% of accumulated length for total SSR motifs). Dinucleotides accounted for the most frequently seen type within tomato and eggplant. Of those imperfect SSR motifs, there were less mono- to tetranucleotide motifs than those seen in perfect SSRs group. Besides, there were larger motifs, which together with penta- to hexa-SSRs, accounted for 22.3% of the accumulated length for overall imperfect SSR motifs (Table 2). It could be discovered that the sum of Di- and Mono- formed the majority of perfect SSRs in all the genomes of Brassicaceae family that were searched and the species, including *C. sativa* and *E. yunnanense*. However, the majority of perfect SSRs in genomes of radish and *C. bursa-pastoris* are primarily formed by di- and trinucleotides. In the *A. thaliana* and *B. vulgaris* genomes, the sum of mono- and trinucleotides was the most frequent type (Figure 1).

**Figure 2:**
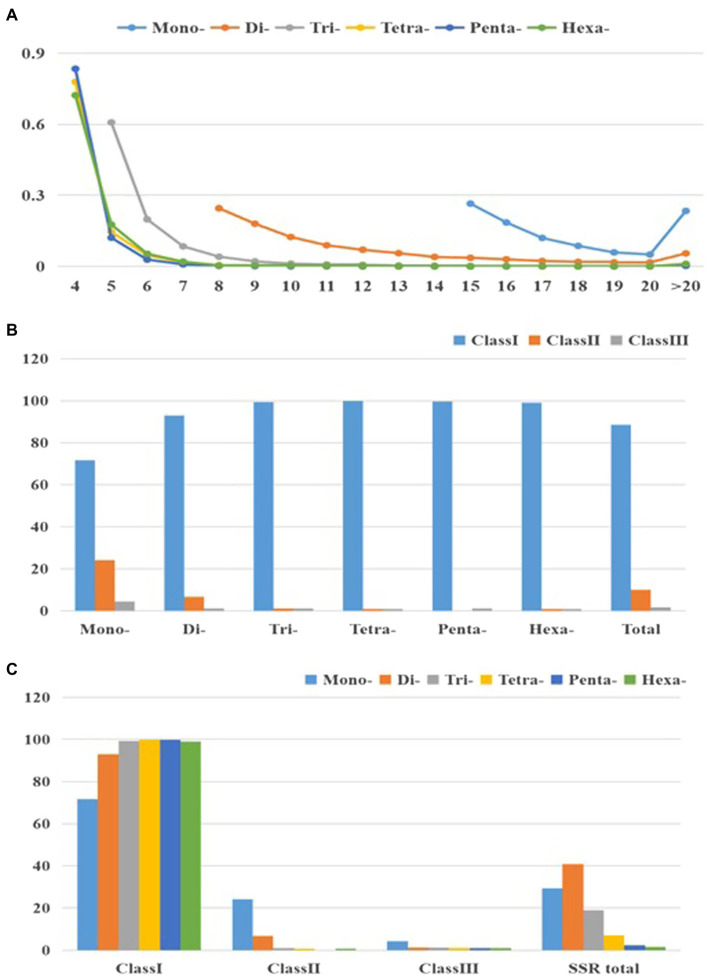
Characterization of perfect SSRs in the cabbage genome. **(A)** The changeable rule from mononucleotides to hexanucleotide motifs. **(B)** The frequency of repeat classes (class I>30nt, class II 20–30nt, class III <20nt). **(C)** The distribution of motif type within each class.

Figure 2 and Supplementary Table S4 present changes in perfect SSR motifs within cabbage genome in terms of repeat unit number. According to our results, larger repeat motifs had significant reduction compared with mono- and di-nucleotide types, among which, tetra- to hexa-nucleotide types experienced the most significant decrease as repeats increased (Figure 2A). Consequently, there were over double dinucleotide repeat units (11.8) relative to hexanucleotide (5.1) and trinucleotide (6.5) ones, and they were about thrice of penta (4.4) and tetra- (4.5) ones (Table 2). According to perfect repeat motif length, we considered 88.6, 9.8 and 1.6% of SSRs as hypervariable class I possibly variable class II and variable class III, respectively (<20, 20–30, ≥30nt, separately; Figure 2B). All the types of nucleotides are members of class I (Figures 2B,C).

### Characterization of SSRs by Classified Type

We classified repeats according to previous description (Jurka and Pethiyagoda, 1995). Therefore, the class AAT of trinucleotide repeats contained (ATA)n, (TTA)n, (TAT)n, (ATT)n, and (TAA)n, and they were the same in terms of diverse reading frames or complementarity. We discovered altogether 331 SSR motif types, and the potential base combinations included mono- (*n*=2), di- (*n*=4) tri- (*n*=10), tetra-nucleotides (*n*=31), together with 80 penta-nucleotide repeat variants and 204 hexanucleotide repeat variants (Table 2).

**Figure 3:**
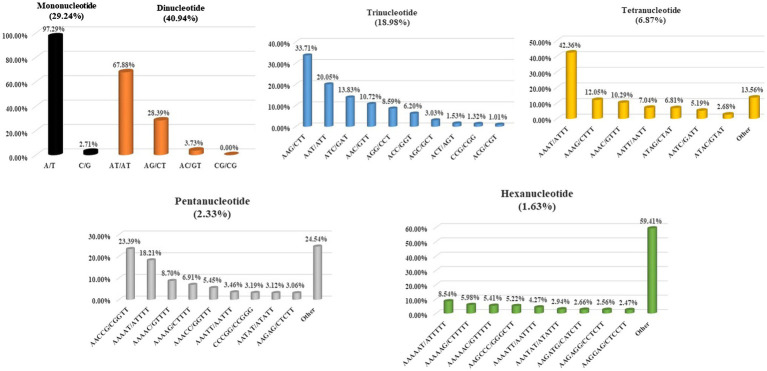
Distribution of the major repeat motifs in cabbage genome.

In this study, the individual repeat motifs for each type of SSR in the cabbage genome were also evaluated (Figure 3 and Supplementary Table S5). The base composition of cabbage SSR motifs is strongly biased toward A and T. The most frequent mono- to hexa-nucleotides motifs were A/T (97.3%), AT/AT (67.9%), AAG/CTT (33.7%), AAAT/ATTT (42.4%), AACCG/CGGTT (23.4%) and AAAAAT/ATTTTT (8.5%). Regarding the distribution of different motifs, the AT repeats were not only the predominant dinucleotides, they were also the most frequent motif in the entire genome, comprising 32.3% of the total SSRs. Alternatively, CG repeats were barely detected. AAT, AAG, AAC, and ATC repeat types occupied the predominant roles in trinucleotide motifs (78.3% in total), while GC-abundant repeats, including CCG, AGC, and ACG, showed low abundances.

Consistently, AT-abundant tetranucleotide motifs, like AAAG, AAAT, AATT, and AAAC occupied the predominant role within cabbage genome (72% totally), whereas motifs AAAAT, AACCG, AAAAG, and AAAAC accounted for 57.0% of overall pentanucleotide repeats. There were just 4 hexanucleotide motif types, including AAAAAG, AAAAAT, AAGCCC, and AAAAAC existing, and the abundance was >5% (Figure 3). The close motif-type distribution was observed among nearly every remaining species detected in the present work (Supplementary Table S6).

### The Distribution of SSRs in the Chromosomes

**Figure 4:**
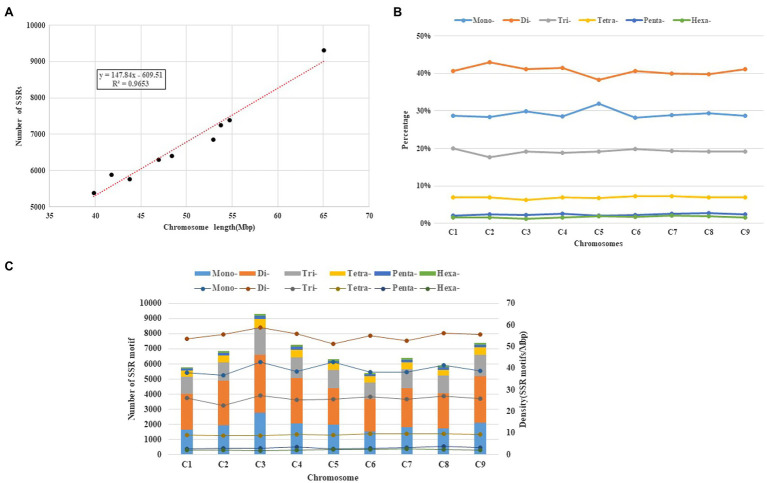
Intra-chromosomal distribution of SSRs. **(A)** Relationship between the SSR number and chromosome length in the cabbage genome. **(B)** The frequency of mono- to hexanucleotide motifs in cabbage chromosomes. **(C)** Number and density of the SSR motifs across nine chromosomes of the cabbage reference genome.

Those SSR loci within cabbage genome discovered were further classified according to the corresponding distribution and motifs in pseudomolecules. There were 7,172 perfect together with 10,414 imperfect SSRs in C1-C9 discovered from pseudomolecules on the whole (Table 3 and Supplementary Table S7). We also predicted the association of chromosome length with SSR number, and a great correlation coefficient *R*^2^=0.9653 was obtained (Figure 4A). There were most SSRs in C3 group (longest linkage; including 13,461 imperfect and 9,311 perfect, 64.98Mbp), whereas C6 group (shortest linkage) had least SSRs (including 7,839 imperfect and 5,393 perfect, 39.82Mbp). Nonetheless, there were great differences in SSR density across diverse chromosomes, which were between 129.55 (C2) and 143.29 (C3) perfect, whereas between 189.79 and 207.16 imperfect SSR/Mbp, separately (Table 3). It was observed that the distribution of motif types within individual chromosomes was very similar to the pattern found over the whole genome, with the mono- and di- repeats observed the most frequently and penta- and hexa- the least (Figure 4B). The number of SSR motifs on each chromosome (C1-C9) ranged from 5,393 (C6) to 9,311 (C3; Figure 4C). Mono- and di- SSRs exhibited maximum variation among linkage groups, with C1 and C5 exhibiting the lowest percentages for di- (38%) and mono- (28%), respectively, and the highest for tri-nucleotides (20%). When diverse motif distributions on the chromosome were considered, the commonly seen mono- to trinucleotides had close proportion to that acquired from the whole-genome. However, the relative contributions of the tetra-, penta- and hexanucleotides varied greatly between different linkage groups (Supplementary Table S5).

**Table 3:** The chromosome-by-chromosome distribution of perfect, compound, and imperfect SSRs.

Linkage groups	Total Mbp	Perfect								Imperfect	
		Mono-	Di-	Tri-	Tetra-	Penta-	Hexa-	Total	SSRs/Mbp	Total	SSRs/Mbp
C1	43.76	1,662	2,351	1,158	397	114	93	5,775	131.97	8,418	192.37
C2	52.89	1944	2,949	1,211	473	164	111	6,852	129.55	10,038	189.79
C3	64.98	2,792	3,832	1786	577	205	119	9,311	143.29	13,461	207.16
C4	53.72	2075	3,008	1,371	503	188	112	7,257	135.09	10,322	192.14
C5	46.9	2007	2,414	1,207	426	130	115	6,299	134.31	8,944	190.70
C6	39.82	1,525	2,190	1,069	393	122	94	5,393	135.43	7,839	196.86
C7	48.37	1846	2,555	1,240	469	160	133	6,403	132.38	9,501	196.42
C8	41.76	1735	2,349	1,132	407	162	107	5,892	141.09	8,512	203.83
C9	54.68	2,127	3,050	1,424	512	177	111	7,401	135.35	10,683	195.37
Total	488.954	18,876	26,425	12,253	4,433	1,505	1,054	64,546	132.01	93,724	191.68

### Gene Context of SSRs

**Figure 5:**
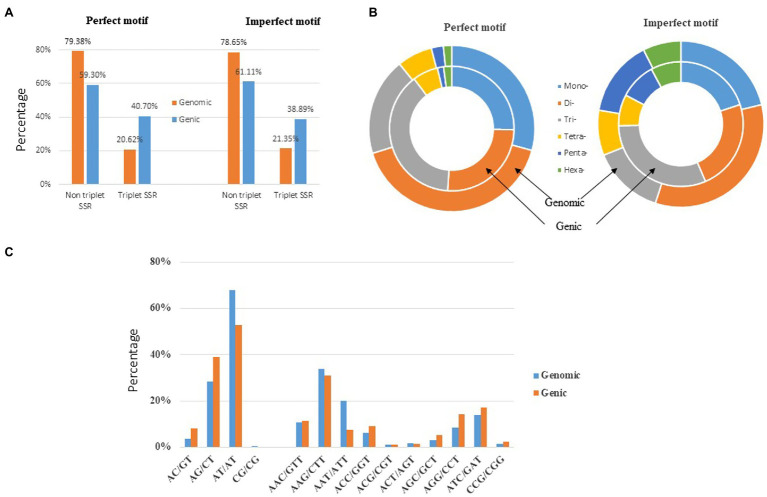
Distribution of microsatellite sizes in the cabbage genome. **(A)** Non-triplet SSR vs. triplet SSR in both perfect and imperfect motifs. **(B)** Distribution of repeat types within perfect and imperfect SSR motifs. **(C)** A comparison between di- and trinucleotide repeats in both the gene space and full genomic region.

**Figure 6:**
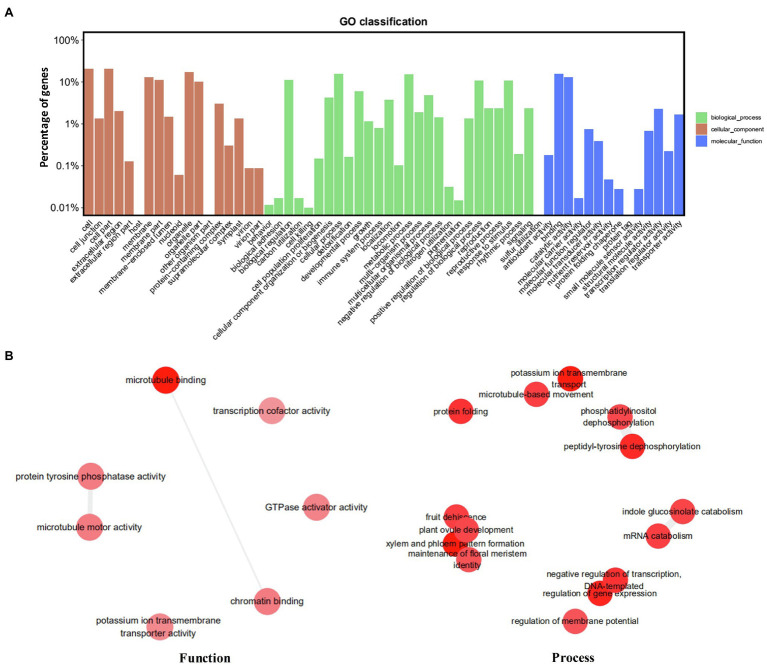
Functional analysis (gene ontology) of cabbage genes containing SSRs. **(A)** GO classification. **(B)** Revigo summary of “biological process” and “molecular function” enriched terms.

The genomic distribution of SSRs was compared with their association with individual genes based on the data from assembled chromosomes of cabbage genome (Gadaleta et al., 2012; Bhattarai et al., 2021). A total of 14,349 perfect SSRs (22.23%) and 18,889 imperfect SSRs (20.15%) were associated with 11,013 (18.15%) and 13,707 (22.58%) genes, respectively (Table 4). It accounts for 18–22% of the entire gene space. It was predicted that such cabbage genes covered altogether 29.38 Mbp; in other words, the density was 138.24 for perfect whereas 181.97 SSRs/Mbp for imperfect motifs, separately, in the gene space. Based on repeat motifs, we divided SSRs distribution on every pseudomolecule (Supplementary Table S8). Figure 5 presents the comparisons of SSR motifs discovered from genomic and genetic sets. We assigned the overall SSRs populations within the gene space and genome as non-triplet repeats (mono-, di-, tetra-, penta-nucleotides) and triplet repeats (tri-, hexanucleotides). As for imperfect (38.89%) and perfect (40.7%) motifs, there were more gene sequences in triplet repeats (Figure 5A). Typically, trinucleotides represented the most frequent type, occupying 38.58% (53.3 SSR/Mbp) for perfect whereas 31.04% (56.49 SSR/Mbp) for imperfect genic SSRs, separately (Table 4 and Figure 5B). The most common dinucleotides were AT/AT. They comprised 21.1% of the total genic SSRs. The most frequent genic SSR motif types were the trinucleotides AAG/CTT (31.0%), ATC/GAT (17.1%), AGG/CCT, and AAC/GTT (Figure 5C). Therefore, we compared a group of SSR genes in the cabbage reference gene space and evaluated the specific gene regulation functions that are frequently present. The genes that contained one or more SSRs were discovered within 60 sub-GO categories (“biological processes” (BP), “cellular components,” (CC) and “molecular function” (MF); Figure 6 and Supplementary Table S9). Over-representation was found for a number of gene families, such as BP in the sub-categories “Xylem and phloem pattern formation” (GO:0010051), “Potassium ion transmembrane transport” (GO:0071805), and “Regulation of gene expression”(GO:0010468); for MF, “Microtubule binding” (GO:0008017) and “Microtubule motor activity” (GO:0003777). No enrichment was observed for CC.

**Table 4:** Variation in repeat length among genic cabbage perfect and imperfect SSRs.

SSR type	Perfect motif			Imperfect motif		
	Count	%	Density(SSRs/Mbp)	Count	%	Density(SSRs/Mbp)
Mono-	3,632	25.31	34.99	3,768	19.95	36.30
Di-	3,697	25.76	35.62	4,456	23.59	42.93
Tri-	5,536	38.58	53.33	5,864	31.04	56.49
Tetra-	951	6.63	9.16	1,535	8.13	14.79
Penta-	229	1.60	2.21	1785	9.45	17.20
Hexa-	304	2.12	2.93	1,481	7.84	14.27
Total/mean	14,349	100.00	138.24	18,889	100.00	181.97

### Development and Validation of Unique SSR Primer Pairs

We also obtained flanking sequences for each SSR motif within cabbage genome, which were adopted to be the targets to design primers. For obtaining specific primer pairs, they were aligned against reference genome of cabbage according to primer selection criteria. Finally, altogether 64,546 primer pairs were obtained (Supplementary Table S10).

This study prepared 1,113 SSR primers (Supplementary Table S11) at random, analyzed them for preliminary verification, and amplified them by 2 DNA templates of cabbage, namely, ‘JSC142’ and ‘JSCJF’. Among the SSR primers, 916 pairs (82.3%) were stably and repeatedly amplified. For better confirming whether sequences that contained polymorphic microsatellites were real and positive, we recovered and sequenced 30 co-dominant segregation segments following T-A cloning. As a result, these sequences conformed to the initial ones, which indicated the high specificity of our prepared SSR primers.

### Genetic Diversity Analysis of Cabbage Genotypes

**Figure 7:**
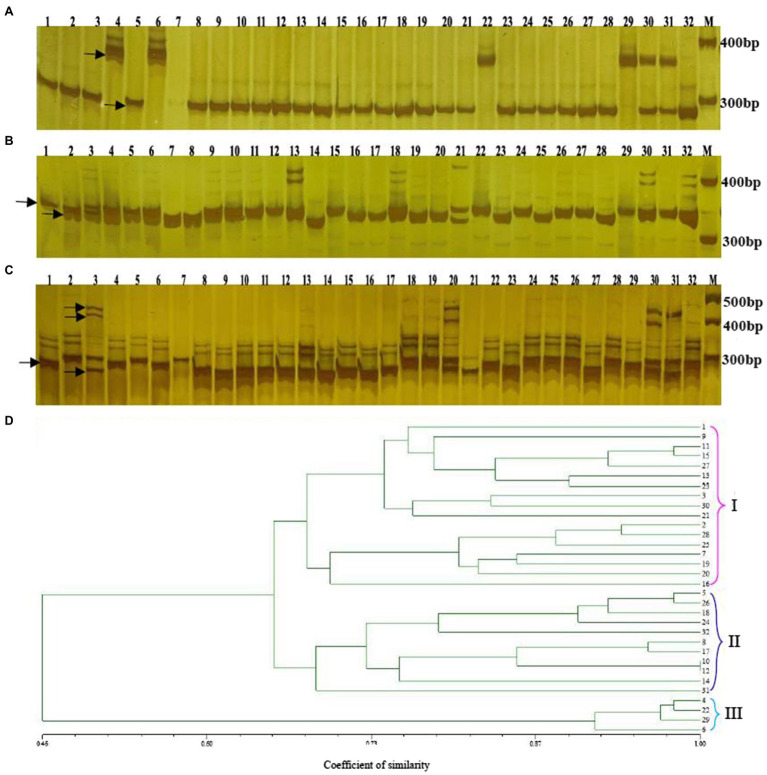
Genetic diversity analysis of 32 cabbage accessions with SSR markers. Amplification of 32 genotypes with the SSR primers Bol-SSR32 **(A)** Bol-SSR24 **(B)** and Bol-SSR12 **(C)** by polyacrylamide gel electrophoresis, and the UPGMA dendrogram of 32 cabbage genotypes based on 32 new SSR markers **(D)** M: 50bp DNA ladder. The genotype name of numbers (1–32) is listed in Supplementary Table S1.

This study prepared altogether 60 possible SSRs to conduct PCR validation by using the PAGE gels. Among them, 32 primers exhibited diacritical polymorphisms across diverse genotypes (Figures 7A–C). For investigating the possibility of using those candidate SSRs to carry out genetic analysis, we chose 32 SSR primers showing polymorphism for assessing genetic diversity for those 32 cabbage cultivars obtained from diverse areas (Supplementary Table S1). We discovered altogether 105 alleles, including 92 (87.67%) polymorphic alleles. On average, there was 2.9 alleles at each locus (range, 1–8). Additionally, the average PIC value was 0.46 (range, 0.14–0.88; Table 5). The sizes of the amplicons for the SSRs markers ranged from 134bp to 273bp. The information of these informative SSR primers is shown in Table 5.

**Table 5:** Genetic diversity analysis with SSR markers developed in cabbage.

Primer name	SSRID	Forward primer (5'-3')	Reverse primer(5'-3')	Expected size (bp)	Tm	Allele no.	PIC
Bol-SSR1	NC_027748.1:3927:SSR1	GTCGTCGTCGGTCATTAGGT	TATCCATCACCCCCTAACCA	200	60	2	0.14
Bol-SSR2	NC_027748.1:4466:SSR2	TCCTCATGGTCCCTTGCTAT	TGATACCTACCCAAGCAGGC	252	60	4	0.31
Bol- SSR3	NC_027748.1:7081:SSR3	GAGACTGAGACAGATCCCGC	TGGCGATGGTAGAGAAGAAAA	237	59	1	0.88
Bol- SSR4	NC_027748.1:8162:SSR4	CTCAGACGGTTGCAGATTCA	TCTCCGTTAACATGGCCTTC	169	60	2	0.24
Bol- SSR5	NC_027748.1:8546:SSR5	TACTAGGCGATGGCAGACCT	TCGTTGAATCCCACCTCTTC	134	60	2	0.16
Bol- SSR6	NC_027748.1:27390:SSR6	ATAAATGATGCGGCCAGAAG	GCAAGGAAGATCCATCGAAA	187	60	7	0.35
Bol- SSR7	NC_027748.1:31848:SSR7	CGAATTCGCTGTCATCTTCA	AGCCACCCGGTGAAACTTTA	229	61	5	0.21
Bol- SSR8	NC_027748.1:38054:SSR8	TTTCCGTAATCGCAAGGAAC	ATGGTCACCGACCTCAACAT	262	60	2	0.22
Bol- SSR9	NC_027748.1:38711:SSR9	GAAAAGCAGCACAACAACGA	CCACAAAACGTGTGCTTGAG	272	60	2	0.45
Bol- SSR10	NC_027748.1:48258:SSR10	CCCTCTCCTTTTTCTTTCGC	ATCTTTGCTGCTTCGGTCTC	238	60	1	0.51
Bol- SSR11	NC_027748.1:50103:SSR11	GTAGGGGTGGTCCAAAAGGT	CTTGGATCCTTCTCCCACAA	197	60	1	0.39
Bol- SSR12	NC_027748.1:51423:SSR12	GCTTTATACGCCGTCTCTCG	TTGAAAAGAGCCGCTCCTAA	186	60	8	0.47
Bol- SSR13	NC_027748.1:56608:SSR13	GTGTTTGAAGGAGAGCCGAG	GAACTAAAAAGAGCGCGTGG	197	60	3	0.42
Bol- SSR14	NC_027748.1:65043:SSR14	GGGAGATTATGGGCCTTCTC	TTGGTGGGATGGATGTGAAT	140	60	2	0.52
Bol- SSR15	NC_027748.1:65170:SSR15	GTACCAAAACGAAAGGCCAC	TGAAGTATCCAGAGGGGCAC	273	60	2	0.63
Bol- SSR16	NC_027748.1:116242:SSR21	CTTTTTCAGTCCGTAAGGCG	GCGGGTTACATCCGAAATTA	252	59	2	0.77
Bol- SSR17	NC_027748.1:104629:SSR17	TGAATGGCAAATTCCACAAA	ACGTTGAGATGGCAGGAATC	251	60	3	0.38
Bol- SSR18	NC_027748.1:105374:SSR18	TTATAGCAATCCCCACAGCC	CCATTGTCCTGGCTTTGATT	211	59	4	0.36
Bol- SSR19	NC_027748.1:105555:SSR19	AATCAAAGCCAGGACAATGG	CTCCTCAAGAGCACACTCCC	190	59	6	0.49
Bol- SSR20	NC_027748.1:108306:SSR20	TCGGCGGTTTCTTTTATTTG	CGTTTGTATTGAACCCAGCA	261	60	2	0.69
Bol- SSR21	NC_027748.1:116613:SSR22	GGCAATTGCACTAAATGACCA	TCTCGAGCTTCCCATCTTTG	256	60	2	0.65
Bol- SSR22	NC_027748.1:117330:SSR24	GATTCTAGTCCGGCGATGAC	AACTGGCCTTAATGGCTTCA	146	59	3	0.41
Bol- SSR23	NC_027748.1:135117:SSR25	GGCAAGGATGGACATGATCT	TTCAGAGGATGGAGAGCGAT	237	59	2	0.33
Bol- SSR24	NC_027748.1:293746:SSR58	CGCTGGACCACTTGTACTGA	CCGGCTAATTTACAGCTCCA	170	60	3	0.48
Bol- SSR25	NC_027748.1:135343:SSR26	TCGCTCTCCATCCTCTGAAT	TTGATTGATTGCTTGCTTGC	185	59	2	0.52
Bol- SSR26	NC_027748.1:137015:SSR29	CTCATCGTCGGGATCATCTT	GAGCAGAGTAGCGGAACCAC	192	60	5	0.23
Bol- SSR27	NC_027748.1:138313:SSR30	ATCCCTCCCCATTTTACCAG	GAGATGGCTAAGCGTCAAGG	134	60	4	0.19
Bol- SSR28	NC_027748.1:142981:SSR35	CTGAGACCAACGTGAGCGT	AAATTGGAGACGAAGGCAGA	231	60	2	0.81
Bol- SSR29	NC_027748.1:154301:SSR36	CACGTGAATTGCTCGTGTTT	GAACGTCGACGAATTTGGTT	226	59	2	0.78
Bol- SSR30	NC_027748.1:155819:SSR37	TCCGAAACTACCCCTCTCCT	CGATTACCTCCTGAAATCCG	155	60	3	0.67
Bol- SSR31	NC_027748.1:161172:SSR38	AAGCCAACCAACTCCCTGTA	TGGATCGATCTAAGCGAAAAA	251	59	1	0.32
Bol- SSR32	NC_027748.1:211682:SSR47	CCATTCGCAGCTGTATTTCA	ACCCACTGATGCATACCTCC	182	59	2	0.74

The genotype data were analyzed *via* NTSYS-2.10e software, and the dendrogram showed that 32 cabbage accessions could be classified into three major clusters with similarity coefficients that ranged from 0.46 to 1.00 (Figure 7D). Clusters I and II included 17 and 11 accessions, respectively. Most of the accessions in these two clusters had different geographical origins, leaf colors, ball shapes, and maturities. In Cluster I, JSC28 presented a high similarity with JSC40. Both originated in China and have green leaves and mature early. JSC37 and JSC111 were divided into a subgroup. Both originated in Europe and are gray-green. JSC3 that originated in Japan and JSCJF that originated in China were assigned to one subgroup, since both have a similar leaf color and ball shape. JSC13 that originated in China, and JSC90 that originated in Japan were also classified as one subgroup, because they had round ball morphology, green leaves, and early maturation. We classified JSC185 originating in Japan and JSC2 originating in China as one subgroup. Although both have a similar leaf color, they have different spherical characteristics and maturities.

In Cluster II, JSC10 that originated in China and JSC168 that originated in the Netherlands were assigned to one subgroup. Both have the same ball shape. They differ in that JSC10 has yellow-green leaves and matures extremely early, while JSC168 has purple leaves and matures late. JSC18 that originated in Japan and JSC43 that originated in China were assigned to one subgroup, and both have green leaves, a round ball shape and mature early. JSC23 and JSC30 were assigned to one subgroup. Despite that they differed in origin, they have the same spherical shape, leaf color and stage of maturity. A wild accession JSCYS, which was collected in the United States, and it has dark green leaves and a non-heading character was assigned to a separate subgroup in Cluster II. Cluster III comprised four accessions. All were collected from China and have the same spherical shape. JSC7 and JSC410 have the same color leaves and state of maturity. JSC12 has gray-green leaves and matures extremely late, while JSC107 is blue-green and matures at a medium stage.

### MCID of Cultivar Identification With SSR Markers

**Figure 8:**
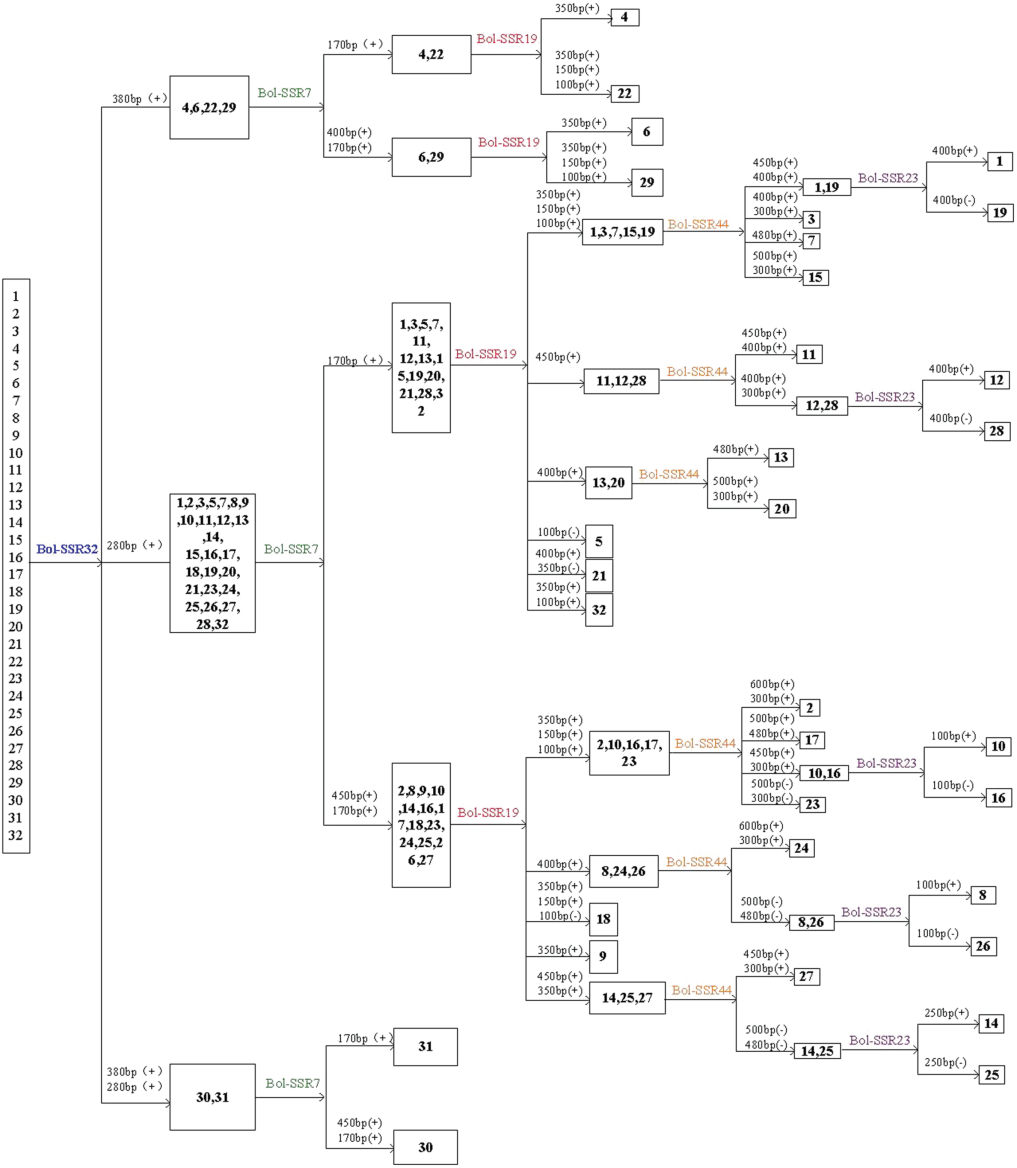
MCID analysis of the 32 cabbage genotypes with the DNA fingerprints of five SSR primers. The number above each horizontal line in the diagram denotes the size of the polymorphic bands used to separate the genotypes following the line. (+) or (−) denotes the presence or absence of the polymorphic band.

This study identified 32 cabbage cultivars by using 5 SSR primers that contain the polymorphic and reproducible bands. Of those 5 primers utilized, the Bol-SSR32 primer was initially selected to identify cabbage genotypes (Figure 8). Based on PAGE analysis, the Bol-SSR32 primer produced 2 polymorphic bands within those 32 cultivars (Figure 7A), and it might be used to classify diverse cabbage genotypes to 3 groups according to with/without the characteristic 280 and 380bp bands. Later, the SSR7 primer was used to separate those cultivars in 3 groups singly or to smaller groups, like JSC142 or JSCYS. Afterwards, we used the rest 3 primers to distinguish cabbage cultivars step by step. Typically, applying the Bol-SSR23 primer helped to separate those 32 cultivars from MCID (Figure 8). We screened several clear polymorphic primers during the course of experiment. It should be highlighted that only the clear polymorphic bands amplified with each primer were accurately used to differentiate the accessions. These indicated that the MCID method used in this study is a valuable and efficient strategy for the identification of cultivars in cabbage.

### Application of SSR Primers to Other Species in the Brassicaceae

**Figure 9:**
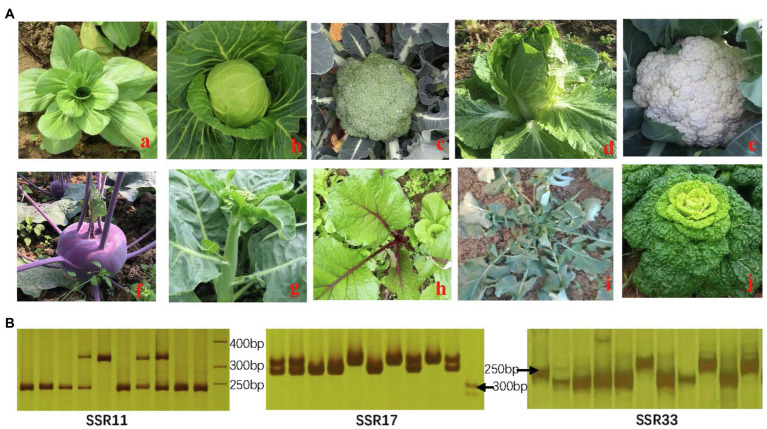
Identification of related species in the Brassicaceae family **(A)** and amplified results by SSR primers **(B)**. (a) *Brassica campestris* subsp. *chinensis* (AA); **(b)** *Brassica oleracea* var. *capitata* (CC); **(c)** *B. oleracea* var. *italica* (CC); **(d)** *Brassica rapa* ssp. *pekinensis* (AA); **(e)** *B. oleracea* var. *botrytis* (CC); **(f)** *B. oleracea* var. *caulorapa*; **(g)** *B. alboglabra*; **(h)** *B. parachinensis*; **(i)** *B. napus* (AACC); **(j)** *B. campestris* L. ssp. *chinensis*.

This study randomly chosen 24 stable and reliable SSR primers for amplification on 10 different species in the Brassicaceae family to identify the potential transferability and availability (Figure 9). In total, 21 of the 24 (87.5%) SSR primers exhibited transferability and applicability to one or more of the 10 related *Brassica* species that were used in this study. Of them, altogether 9 primers exhibited different and stable bands among those 10 species, demonstrating the reliability and applicability of our identified SSR markers for cabbage in certain relevant Brassicaceae family members.

## Discussion

SSR markers have been deemed as the promising candidates to conduct genetic mapping and diversity analyses on crop species because they are specific and highly conserved (Yang et al., 2015). More and more articles have revealed that it is a highly effective and low-cost way to identify SSR markers on the basis of NGS. We collected and identified the genome-wide data of several cruciferous crops, including *B. oleracea*, to develop more SSR markers. A total of 64,546 perfect and 93,724 imperfect SSR were identified. Functional markers were employed in the genetic diversity analysis, to identify the cultivars among 32 different genotypes of cabbage and in the availability analysis across 10 relatives in the cruciferous crops.

The distribution of SSRs was examined within 12 genomes in the Brassicaceae family. Besides, it was discovered that genome size showed positive correlation with SSR motif number discovered after comparing the 12 species. Several reports showed that species that possessed larger genomes typically display a lower SSR density (SSRs/Mb; Morgante et al., 2002; Portis et al., 2018). Nonetheless, the different genomic sizes will result in different microsatellite repetition levels, but SSR density is not associated with genome size (Behura and Severson, 2014; Portis et al., 2016). In this study, the three species *B. rapa*, *C. bursa-pastoris* and *B. vulgaris* were found to have larger genomes and exhibit a lower density of SSR. However, the species *B. nigra* and *E. yunnanense* are exceptions. Their genomes sizes are 402.1 and 415.4 Mbp, but their microsatellite densities are comparable to those found in *B. napus* that has a genome twice as large. The density of perfect microsatellites in the *B. oleracea* genome is the fourth highest observed within the *Brassica* family, even though it is similar to those detected in *B. nigra* and *E. yunnanense*. Consequently, SSRs are highly enriched and abundant within Brassicaceae, making them the attractive molecular markers to carry out genetic analyses of Brassicaceae (Lv et al., 2017).

In this study, we compared the classified types of SSR motifs among all the species in the Brassicaceae family. Variation in the selective constraint on sequence repeats could differ among the SSR motifs. Overall, cabbage had the second greatest number of motif types compared with the other members in this family. It is still unknown about whether such heterogeneity is associated with the species genomic evolution or complexity.

Dinucleotides were considered as the most common type in eggplant and tomato. In our study, although dinucleotides are the most common repeats in the cabbage genome, trinucleotides prevail in the gene space, which is analogous to those of other species (Cavagnaro et al., 2010). In contrast, tri- and hexanucleotides are the most common type in the gene space of eggplant genome. The reason could be attributed to negative selection against frameshift mutations in the coding regions, and because of the mutation pressure combined with possible and positive selection for specific single amino acid stretches, the trinucleotides have enhanced their frequency in the coding portion. Generally, AT-rich motifs occur more often in dicotyledons. It has been reported that AT-rich repeats are widespread in dicotyledonous but not in monocotyledonous species, and the difference between them may be partially accounted for by the nucleotide composition of their genomes. The monocots have a GC content of 43.7% compared with one of 34.6% in the dicots (Portis et al., 2018). We found similar results in the cruciferous crop genomes. However, the classified motif types were not completely identical within each species, which was shown by comparisons among several genomes in this study. The most common dinucleotides in this study were AT/AT, and the most frequent genic SSR motif types were the trinucleotides AAG/CTT. Similar patterns of motif distribution have been found in other species. For example, several studies reported that AT/AT repeats appear to be typical in non-transcribed regions, and AG/CT prevail in gene sequences, while AC/GT and CG/GC repeats are the least frequent dinucleotides in both genomic and gene sequences (Cavagnaro et al., 2010; Portis et al., 2016). GC-rich motifs showed a strong bias in their distribution in cabbage gene sequences, most notably for mono-, di- and tri. For example, a GC content of only 14% was discovered in the whole genomic trimeric SSRs, whereas the trinucleotide SSRs in genes had a GC content of 43%. Genic SSR markers show a higher efficiency among various species when compared with non-coding microsatellites, promoting their application as anchor markers suitable for comparative genetics analysis (Varshney et al., 2005). On the contrary, coding SSRs experience an increased selection pressure; as a result, only insufficient polymorphism degree can be provided for distinguishing the varieties with close relations. Nonetheless, the genetic SSRs of cabbage can offer a decreased number of possibly variable SSRs, because of the decreased corresponding repeat number compared with that within the whole genome; typically, among the SSRs, 63.04% contained ≤10 repeats, while just 8.43% contained ≥20 repeats. Over-representation was found in several subcategories, such as BP and MF, but no enrichment was observed. In previous studies, SSRs are reported to occur in certain gene functions within eggplants, globe artichoke and *Medicago truncatula*, while transcription factors (TFs) constitute a distinct gene class containing SSRs (Portis et al., 2016, 2018; Min et al., 2017). In addition, TFs carrying SSRs have also been suggested to have critical functions, and their association with species diversity in Brassicaceae family should be clarified.

Longer repeats have been reported to show a lower abundance level within each class. In certain species, SSR frequency decreases as the repeat number increases, like globe artichoke and *Capsicum* (Cheng et al., 2016; Portis et al., 2016). For instance, SSRs that contain ≤10 repeats take up approximately 50% of the whole SSR number, whereas SSRs that contain >20 repeats only occupy <10%. According to our results, longer repeat motifs had significantly greater decreasing amplitude than mono- and di-nucleotide ones, among which, tetra- and hexa-nucleotide motifs exhibited the greatest decline in their frequencies as the repeat number increased. Some microsatellites were often found between neighbor genes that were reported to possibly be involved in gene regulation (Gao et al., 2013; Sawaya et al., 2013). Such microsatellite hotpots were also observed in this study, although they were mostly owing to long stretches of compound microsatellites. However, since most of the highly mutable loci are compound microsatellites that are composed of two or more repeated motifs, they could be exploited as putative highly polymorphic markers.

However, genic SSRs have been demonstrated to exert an important role in gene expression and function in both humans and plants, which stand for a class of ‘functional markers’ in transcripts. They are also known as microsatellite instability (MSI), and MSI is known to enhance with plant development in *A. thaliana* (Golubov et al., 2010; Nelson et al., 2013). In previous studies, the occurrence of SSRs within specific gene functions has been found, and transcription factors form a significant class of genes that contain SSRs. Furthermore, the important role of transcription factors that possess microsatellites has been pointed out, and the relationship between this tendency and the species diversity of the Brassicaceae merits further study (Li et al., 2004).

To date, functional genetic markers, including SSRs, have progressively become a powerful approach to obtain insight into genetic studies owing to their multi-allelic detection, reproducibility and high cross-species transferability (Thiel et al., 2003; Taheri et al., 2018). With the emergence of NGS technology, the large-scale development of SSR markers based on genome-wide analysis directly or indirectly promotes the rapid development of marker-assisted breeding. A substantial number of SSR markers have been widely recognized in a variety of plants, including black pepper, pepper, pear, bitter gourd, bread wheat, *Camellia sinensis* and eggplant, by the analysis of genome-wide sequence data generated (Cui et al., 2017; Liu et al., 2018; Portis et al., 2018; Xue et al., 2018; Kumari et al., 2019; Uncu, 2019; Ahmed et al., 2020). In this study, a large number of SSR primer sets were the first ones to be comprehensively and successfully designed from the whole genome of cabbage, which is specific to previous studies. Many primers are able to amplify certain bands, whereas some can amplify the significantly greater bands, possibly because that the repeat number is changed or there is one small intron between primer pairs. Moreover, non-PCR fragment-producing primers might be associated with the existing huge introns or null alleles or the primer pair design among the splice sites.

Some recent articles have reported that SSR markers have been applied in the diversity and fingerprinting analyses within some plant species. According to our results, the whole-genome-based SSR markers showed high efficiency in distinguishing 32 cabbage species, and their distributions were not totally decided by the corresponding geographical sources, conforming to our prior works. According to the obtained results, the SSR markers extracted from the genome data of *B. oleracea* L. were suitable and served as excellent markers to distinguish cultivated landraces from wild ones. MCID is a new strategy that is more practical, economical, and effective at identifying plant cultivars with fewer primers, and the proposed method creates a recordable and readable flow chart, enabling the much easier identification of cabbage cultivars.

In addition, the genome-based genetic markers produced in this study are highly conservative and transferable from cabbage to some related cruciferous species, which is consistent with the results of research on cereals and the Leguminosae, Cucurbitaceae, and Rosaceae. However, the novel SSR markers developed with a relatively high level of transferability and availability will be conducive to advancing the investigation of comparative mapping analyses in the Brassicaceae family. In brief, the SSR markers developed based on the WGS data in this study have polymorphism, repeatability, and transferability and will become an important tool for genetic mapping, germplasm identification and genetic diversity analysis of cabbage and its related species in the future.

## Conclusion

In this study, a large number of potentially variable SSRs have been identified in cabbage. We identified 64,546 perfect SSR motifs and 93,724 imperfect SSR motifs in the 0.5Gb of the cabbage genomic sequence, which was mined using a whole-genome bioinformatics survey. The cumulative length of full collection of cabbage SSRs was 1.4Mbp, which comprises 0.29% of the assembled genome. Considering all Brassicaceae family members, the genome size was found to be positively associated with the number of SSR motifs identified. Dinucleotide sequences were the most common type in all cabbage SSR motifs, comprising 0.64Mbp. As expected, microsatellites are ubiquitously distributed, and we detected a higher content of SSR repeats for longer chromosomes, as well as the homogeneous distribution of SSRs. Such innate characteristics of SSRs render them the suitable markers. Additionally, those 32 informative SSR markers chosen were adopted for determining the 32 cabbage genomes for their genetic diversity. Cultivars were efficiently identified by using the new strategy designating the manual diagram for identifying cultivars, and 5 Bol-SSR markers were utilized to distinguish 32 cabbage accessions. In addition, we also verified the transferability and availability of such SSRs based on additional 10 species belonging to Brassicaceae family. These results suggest that the genomic SSR markers that have been developed have considerable potential value in advancing cabbage research, including genetic mapping, MAS, and comparative genome analyses.

## Data Availability Statement

The original contributions presented in the study are included in the article/Supplementary Material, further inquiries can be directed to the corresponding authors.

## Author Contributions

YX performed the data analysis and drafted the manuscript. MX, AZ, and LS conducted the validation of experiments and data analysis. WL and AZ contributed powerful analytical tools. AZ and JY helped with the revise of the manuscript. YX and AZ conceived and designed the research. All authors read and approved the final manuscript.

## Funding

The study was supported by the Natural Science Foundation of Jiangsu Province (No. BK20190262).

## Conflict of Interest

The authors declare that the research was conducted in the absence of any commercial or financial relationships that could be construed as a potential conflict of interest.

## Publisher’s Note

Publisher’s NoteAll claims expressed in this article are solely those of the authors and do not necessarily represent those of their affiliated organizations, or those of the publisher, the editors and the reviewers. Any product that may be evaluated in this article, or claim that may be made by its manufacturer, is not guaranteed or endorsed by the publisher.

## Footnotes

^1^ http://kofler.or.at/bioinformatics/SciRoKo

^2^ http://pgrc.ipk-gatersleben.de/misa/primer3.html

^3^ http://www-genome.wi.mit.edu/genome_software/other/primer3.html
